# Crown Ether Copolymerized Polyimide Film: Enhanced Mechanical, Thermal Properties and Low Dielectric Constant under High Frequency

**DOI:** 10.3390/polym16091188

**Published:** 2024-04-24

**Authors:** Heming Li, Xinming Wang, Ziyang Ding, Weiguo Gao, Yan Liu, Ke Ma, Zhizhi Hu, Yongqi Wang

**Affiliations:** 1 School of Chemical Engineering, University of Science and Technology Liaoning, Anshan 114051, China; lhm900308@163.com (H.L.); wangxm0410@163.com (X.W.); dzy15604294797@163.com (Z.D.); gao990322@163.com (W.G.); 18523501103@163.com (Y.L.); 2 Oxiranchem Holding Group Co., Ltd., Liaoyang 111003, China; 3 Liaoning Agricultural Technical College, Yingkou 115009, China

**Keywords:** crown ether, polyimide, dielectric constant, high frequency

## Abstract

Polymer materials with a low dielectric constant and low dielectric loss have the potential to be applied to high-frequency signal transmissions, such as mobile phone antennas and millimeter wave radars. Two types of diamines, 4,4′-diamino-p-tetraphenyl (DPT) and crown ether diamine (CED), were prepared for ternary copolymerization with BPDA in this study. Cross-links with molecular chains were formed, increasing molecular chain distance by utilizing rings of CED. The MPI films exhibit a good thermal performance with the increase in CED addition, with Tg > 380 °C and CTE from −4 × 10^−6^ K^−1^ to 5 × 10^−6^ K^−1^. The Young’s modulus can reach 8.6 GPa, and the tensile strength is above 200 MPa when 5% and 7% CED are introduced. These MPI films exhibit good mechanical performances. The dielectric constant of PI−10% film can go as low as 3.17. Meanwhile, the relationship between dielectric properties and molecular structure has been demonstrated by Molecular Simulation (MS). PI molecules are separated by low dielectric groups, resulting in a decrease in the dielectric constant.

## 1. Introduction

Dielectric materials generate induced charges and weaken the electric field in the external electric field. The dielectric constant (D_k_) is the ratio of the external electric field of the medium in a vacuum to the electric field in the final medium. The dielectric constant reflects the polarization ability of a medium under the action of an external electric field [1,2,3]. In alternating electric fields, there exists a certain phase angle (i.e., phase difference) between the current phasor and voltage flowing through the dielectric due to the hysteresis effect of dielectric conductivity and polarization. The tangent value of a phase angle is called the loss factor. The loss factor can reflect the power loss of the dielectric or signal attenuation ability of insulation material. The larger the loss factor, the stronger the attenuation ability [4,5,6].The rapid development and widespread application of contemporary electronic information technology, especially microelectronic circuits, require electronic devices to develop in the lightweight direction. However, the higher dielectric constant of dielectric materials can lead to signal transmission slowdown, while the higher loss factor can cause the signal to be converted into thermal energy and lost during transmission [7,8,9,10]. The power loss in the dielectric inter-layer is required to be reduced to ensure the stability and safety reliability of electronic devices [11,12]. Therefore, research into and development of low dielectric constant (D_k_) materials have become urgent priorities.

PI films are widely used in the electronic and electrical industry due to their excellent dielectric, mechanical, and thermal properties. The intrinsic dielectric constant of PI is usually around 3.5, while microelectronic devices and large integrated appliances require the dielectric constant of insulation layer material to be less than 3.2. Common methods of reducing the dielectric constant of PI include introducing fluorinated groups, large volume substituents, introducing flexible connecting units, porous structures, and non-polar inorganic nanomaterials [12,13,14]. The cost of reducing the PI dielectric constant by introducing fluorinated groups is relatively high, and it will also reduce the mechanical properties, adhesive properties, and heat resistance of PI. Introducing large volume substituents/flexible structures into PI generally requires the synthesis of novel diamines or dianhydride monomers, and the reaction process is relatively complex. D_k_ can also be reduced by introducing porous structures. This method costs less, but the size of pores is difficult to control and can easily make the material brittle. Fluorine atoms are able to effectively reduce the dielectric constant of material due to their small polarizability and high electronegativity [15]. Furthermore, the introduction of bulky substituents can increase the distance between polymer chains, increasing the free volume and decreasing the dielectric constant. This fluorine adding method may require the synthesis of new monomers, increasing the complexity and cost of PI material preparation. Meanwhile, fluorine addition may adversely affect the mechanical properties of PI material, as the fluorinated groups may weaken the interaction force between PI polymer chains. Flexible connecting units, such as ether-chains and alkyl-chains, can improve the flexibility of PI material while reducing the dielectric constant. These flexible connecting units act as “buffers” in the PI molecular structure, absorbing part of the mechanical stress and improving the toughness of the PI material. Porous structures can also be used to create tiny cavities in PI material to reduce the overall density and dielectric constant [16]. However, it is difficult to control the cavity size and pores distribution to avoid affecting the overall strength of PI material. Low-polar inorganic nano-materials, such as silica or boron nitride nanoparticles, can be introduced to reduce the dielectric constant of PI materials [17,18,19]. These nano-materials can not only reduce dielectric constant, but also improve the thermal stability of PI materials through their high thermal conductivity. However, it is hard to achieve good nanofiller dispersion due to large interface energy differences between the polymer matrix and inorganic nanomaterials. Inorgani nanomaterials often require surface modification to improve their dispersion and compatibility in the polymer matrix.

Crown ether (CE) is a class of macromolecular heterocyclic complexes with—-CH_2_-CH_2_-O—as the repeating units [20,21,22,23]. It can be served as a host molecule and form supramolecular host−guest inclusion complexes with guest molecules containing alkali, alkaline earth metal ions, and amino or ammonium salts [24,25]. CEs are nested on guest polymers main chains, which increase the molecular size of polymers in three-dimensional directions perpendicular and increase the free volume of the matrix [26,27,28]. In order to achieve a good balance between the mechanical−thermal performance and dielectric properties of the material, crown ether diamine (CED) molecules can be introduced into the PI matrix and copolymered with other monomers to produce cross-links [29,30,31,32]. The method of crown ether diamine (CED) molecules copolymerization is an effective strategy to reduce the dielectric constant. Meanwhile, the introduction CED molecules can not only reduce the dielectric constant but also improve the mechanical properties and thermal stability of PI material in a certain extent, achieving a balance between the dielectric properties and other properties. In addition, the performance of modified PI material can be further optimized by adjusting the type and addition amount of CED, making specific PI application more in line with the needs.

## 2. Experimental Section

### 2.1. Materials

DB18-CE-6, 1,4-phenyldiboronic acid, tetra (triphenyl phosphine) palladium (0) (Pd[P(C_6_H_5_)_3_]_4_), 4-bromoaniline, and Pd/C were purchased from Aladdin Biochemical Technology Co., Ltd. (Shanghai, China) Acetone (AC), N,N-dimethylacetamide (DMAc), nitric acid (HNO_3_), tetrahydrofuran (THF), dimethyl sulfoxide (DMSO), Diphenyltetraacetic anhydride (BPDA), and anhydrous ethanol were obtained from China National Pharmaceutical Group Chemical Reagent Co., Ltd. (Beijing, China). Dianhydride was dried in a vacuum at 125 °C for 24 h before sample preparation. All other reagents were obtained from Sinopharm Chemical Reagent Co., Ltd. (Beijing, China) and used as received.

### 2.2. Characterization

Fourier transform infrared (FT-IR) spectras were recorded on a Bruker Tensor 27 spectrometer. Wide-angle X-ray diffractions (WA-XRD) were measured on a Rigaku SmartLab X-ray diffractometer. Thermogravimetric analysis (TGA) was performed by using a TG209F1 Libra thermal analyzer (NETZSCH, Bavaria, Germany) at a heating rate of 20 °C/min, from 50 °C to 900 °C, under nitrogen gas. Dynamic mechanical (DMA) spectral analysis was performed by using a DMA Q800 analyzer (TA, New Castle, DE, USA) under tensile conditions with a 0.01 N preload force, 20 mm amplitude, and 125% force trajectory at 1 Hz frequency, as well as a 10 °C/min heating rate. Thermal mechanical analysis (TMA) was measured by using a TMA Q600 analyzer (TA, New Castle, DE, USA) to test the preloading force at 0.05 N. The thermal expansion coefficients (CTE) of PI films were also measured by using a TMA Q600 analyzer at a 10 °C/min heating rate from 50 °C to 390 °C. The dielectric constant and dielectric loss of PI films were measured at 10 GHz by using a QS40A dielectric constant and dielectric loss tester. The sample films were standardized in 35 μm–45 μm thickness by using a coating mechanism (MSK-AFA-H200A, Dalian, China). The size of all samples in the dielectric tests was 10 mm × 120 mm.

### 2.3. Synthesis of Diamine Monomers and Preparation of CED-PI Film

#### 2.3.1. Preparation of 4,4′-Diamino-p-Tetraphenyl (DPT)

**Scheme 1:**
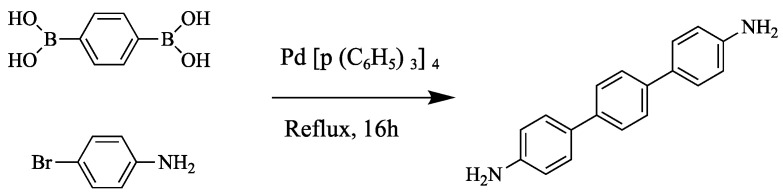
The polymerization of DPT diamine.

A rigid non-planar-conjugated diamine DPT monomer was prepared by a Suzuki cross coupling reaction, as shown in Scheme 1. Additionally, 4,4′-biphenyldiboronic acid (4.986 g) and 4-bromoaniline (8.121 g) were added into 1,4-dioxane solution and stirred at room temperature for 10 min. K_2_CO_3_ and Pd[p(C_6_H_5_)_3_]_4_ were added into the mentioned mixture and then deoxygenated three times with a nitrogen atmosphere at 65 °C for 16 h. The filter cake was washed until colorless. DMSO recrystallization was used to obtain brownish yellow crystal 4,4′-diamino-p-tetrabenzene (DPT, 11.625 g, purity of 99.8%) [33,34]. The Hydrogen spectrum of DPT was shown in Figure S1.

#### 2.3.2. Preparation of Crown Ether Diamine (CED)

**Scheme 2:**
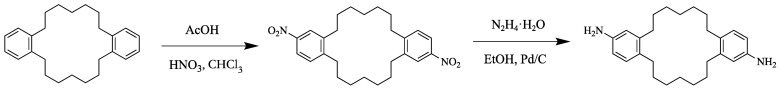
The polymerization of CED diamine.

DB18-CE-6 (10.81 g) was added into a mix solution of HNO_3_, AcOH, and CHCl_3_. The mixture was heated at 50 °C and stirred for 5 h to obtain nitro intermediate. Then, the obtained intermediate, EtOH, N_2_H_4_·H_2_O and Pd/C were added into a flask, and heated at 50 °C for 2 h stirring to obtain crown ether diamine (purity of 99.8%), as shown in Scheme 2 [35,36,37]. The Hydrogen spectrum of CED was shown in Figure S1.

**Scheme 3:**
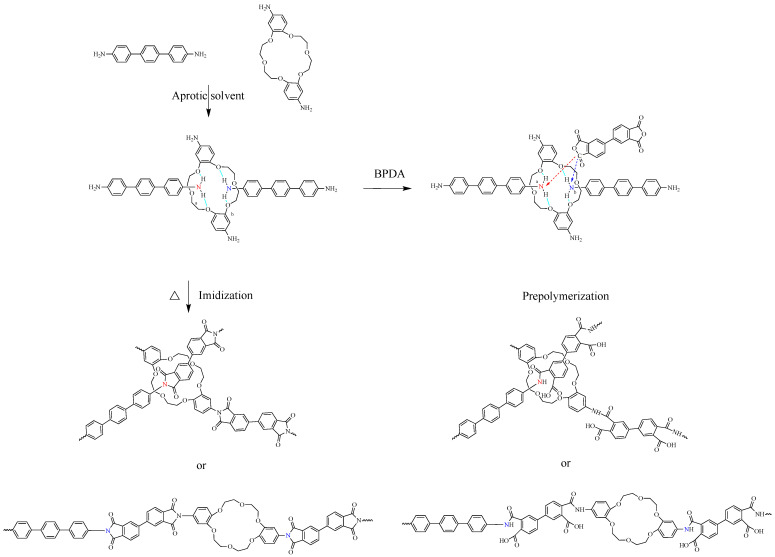
The polymerization of DPT/CED-BPDA MPI film.

Preparation of PI copolymerized film; the preparation process is shown in Scheme 3. The polymerization experimental formula is shown in Table 1. The molar ratio of DPT/dianhydride/CED is 1:1:0.1–0.4. The PI/CED host guest inclusion films were prepared according to the conventional two-step thermal method, as mentioned in our past work [38,39].

## 3. Results and Discussions

### 3.1. Chemical Structures

**Figure 1:**
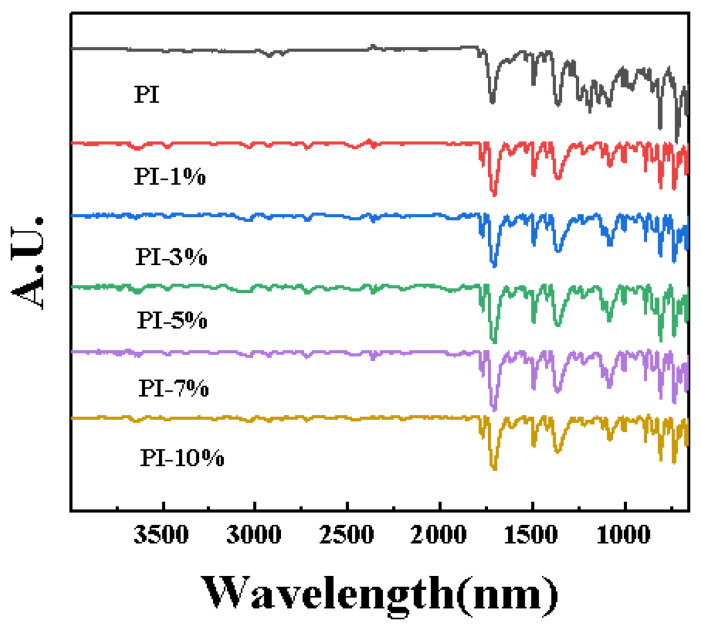
FTIR spectra of PI/CED copolymerized films, PI, PI−1%//PI−10%.

The chemical structures of PI/CED copolymerized films were identified by FTIR, as shown in Figure 1. Asymmetric and symmetric stretching vibration absorption peaks of carbonyl groups (C=O) in an imine ring structure were observed at 1785 cm^−1^ and 1720 cm^−1^, respectively. The stretching vibration characteristic peak of benzene rings was displayed at 1500 cm^−1^. C-N-C bending and tensile vibrations in imide rings were observed at 720 cm^−1^ and 1376 cm^−1^, respectively. The stretching and vibration absorption characteristic peaks of the NH_2_ structure were observed to disappear at 3449 cm^−1^ and 3365 cm^−1^ in PAA solution. These characteristics indicated that the acylation of PI/CED copolymerized films has been completed [40,41].

The characteristic absorption of C-C stretching peak, Ar-O-R stretching peak, and C-O-C stretching peak can be observed at 2942/2853 cm^−1^, 1254 cm^−1^, and 1124 cm^−1^ in the PI−1%, PI−3%, PI−5%, PI−7%, and PI−10% films. These results confirmed the formation of network like composite structures between PI macromolecules and CE molecules, proving that there are crown ether presence groups in PI films [42,43,44,45].

### 3.2. Optical Properties

**Figure 2:**
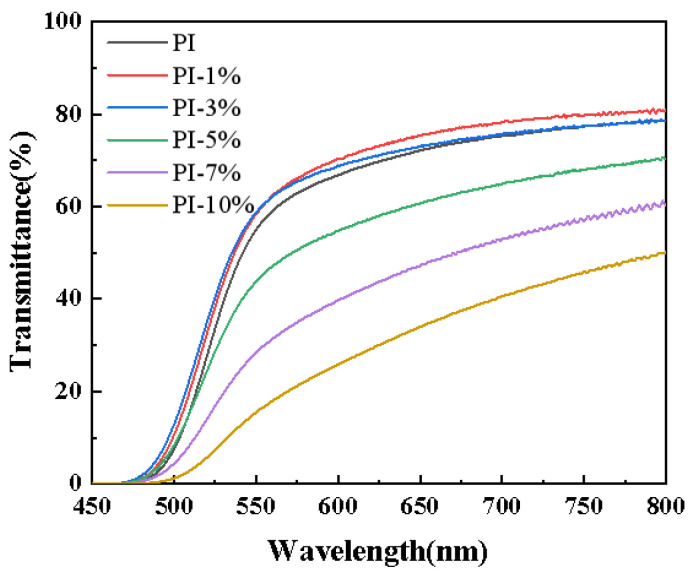
UV-visible spectra of PI/CED copolymerized films, PI, PI−1%//PI−10%.

In Figure 2, the cut-off wavelength of PI, PI−1%, PI−3%, PI−5%, PI−7%, and PI−10% are 483 nm, 479 nm, 476 nm, 470 nm, 468 nm, and 464 nm, respectively. The length of a conjugated system in a PI molecular chain is essential for determining the light-absorption ability. The longer the conjugated system length, the longer the wavelength absorbed by PI material. An energy reduction is required for electrons to move from ground state to excited state, allowing PI material to absorb longer wavelengths of light. The gradual reduction in cut-off wavelengths indicates that the conjugate system is shortened due to the increasing molar content of CEDs. With the increase in CED proportions, the free volumes increase within the PI molecule, reducing the interactions between the molecular chain segments. The optical absorption band of prepared PI films moves towards a shorter wavelength (blue-shift). This blue shift usually indicates that the molecular structure becomes looser or more spaced.

### 3.3. Thermal Properties

**Figure 3:**
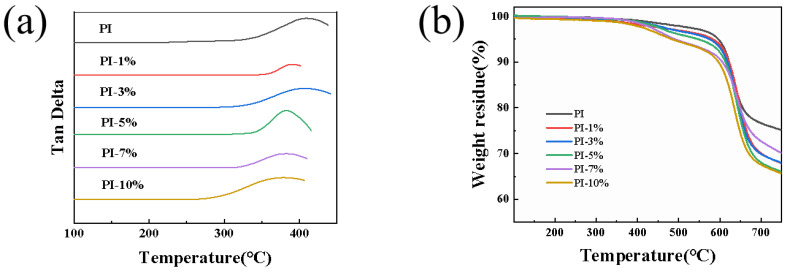
(**a**) DMA curve of polyimide, PI, PI−1%//PI−10%. (**b**) DTG curve of polyimide, PI, PI−1%//PI−10%.

Figure 3a,b and Table 2 show that the PI films have preferable thermal stability, with their Tgs all being above 350 °C. The Tgs for PI, PI−1%, PI−3%, PI−5%, PI−7%, and PI−10% were 409 °C, 391 °C, 407 °C, 383 °C, 382 °C, and 380 °C, respectively. The Tgs values were gradually decreased as the molar content of the crown ethers increased. The introduction of crown ethers may weaken the preexisting hydrogen bond within PI molecular chains. The original mode of molecular interaction was changed by oxygen atoms contained in the crown ethers and the surrounding PI molecules changing. The CED’s flexible chain segments interact differently to inter-molecules from the rigid DPT segments, which may weaken the overall PI polymer network chains. The introduction of the crown ether increased the flexibility of the PI polymer chains. Because the inherent flexibility of a crown ether structure is greater than a rigid terphenyl structure, large molecular movement of the entire polymer chains can be moved at lower temperatures. The Tg value decreased as CEDs increased, as the C-O-C bonds in crown ether rings are more easily broken. Meanwhile, the extricating crown ethers created a significant steric hindrance in PI molecular chains, leading to a decrease in inter-molecular forces and making molecular thermal motion easier [46,47]. PI polymer chains sliding may occur due to the macromolecular cyclic structure of crown ethers, resulting in a slight decrease in Tg values [48].

As shown in Figure 3b, the T_d5%_ values of PI, PI−1%, PI−3%, PI−5%, PI−7%, and PI−10% were 596 °C, 582 °C, 575 °C, 547 °C, 483 °C, and 483 °C, respectively. The T_d10%_ values were 622 °C, 622 °C, 618 °C, 613 °C, 605 °C, and 596 °C, respectively. The Rw 750 were 75.12%, 67.87%, 67.94%, 66.00%, 70.07%, and 65.66%, respectively. PI (no CED introduction) has the highest thermal stability, as the biphenyl structure brings high thermal stability. Thermal stability decreased with the increase in CED addition. The specific macromolecular ring structure of CED occured interactions with other molecular, leading to a decrease in thermal stability of the prepared PI films at high temperatures. The TG-DTG curves of all PIs films exhibited extremely similar thermogravimetric change. These PI films exhibited high thermal stability which exhibited main weight loss above 590 °C, with residual carbon content exceeding 60% at 750 °C. The high thermal stability is due to the strong stability of the biphenyl structure. This is mainly attributed to the steric hindrance of crown ethers. However, as the increase in CED, the copolymerized network structure leads to cross-linking of PI molecular chains, which relatively improves their thermal stability. Furthermore, the combination of CEs and dianhydride also prevents CE molecules volatilizing from PI films, and increased its stability [41,49]. In addition to direct thermal oxidative degradation, the presence of crown ethers promotes specific breakage and crosslinking reactions. The effects of these reactions may vary with different CED contents, resulting in fluctuations between Rw750 values. The compositions of PI films become more complicated as crown ether contents increase. In a word, the crown ether addition may affect thermal stability by its own content increase, and the interaction with PI backbone and different contents of crown ether molecules also affect thermal stability. This complexity may lead to the subtle variation in Rw 750 between different samples.

**Figure 4:**
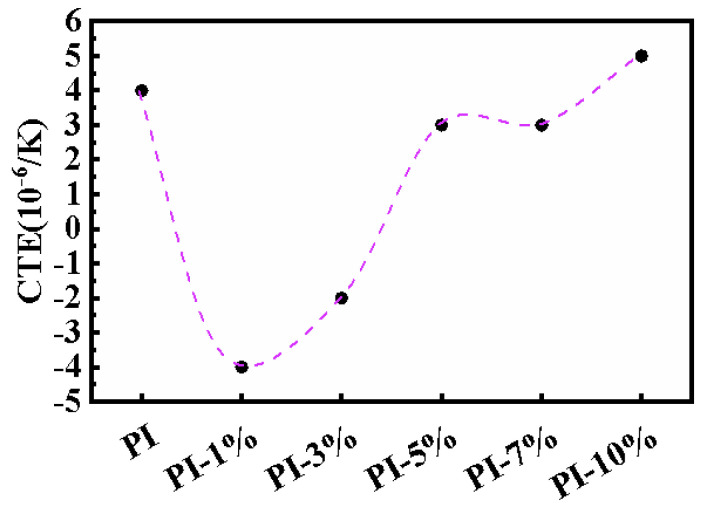
CTE of polyimide film, PI, PI−1%//PI−10%.

Figure 4 and Table 2 show the coefficient of thermal expansion (CTE) of origin PI and PI/CED copolymerized films. It can be observed that the CTE of origin PI was low to 4 × 10^−6^ K^−1^. This can be attributed to the planar-conjugated rod-shaped biphenyl structure in BPDA, which exhibited strong intermolecular forces and low steric hindrance [50,51]. With the addition of CED, The CTE of PI−1%, PI−3%, PI−5%, PI−7%, and PI−10% were −4 × 10^−6^ K^−1^, −2 × 10^−6^ K^−1^, 3 × 10^−6^ K^−1^, 3 × 10^−6^ K^−1^, 5 × 10^−6^ K^−1^, respectively. The introduction of CEDs changes the interaction between PI molecular chains. CEDs enhance the inter-molecular forces by forming hydrogen bonds, which perform better in regard to dimensional stability under the action of thermal energy. The enhanced inter-molecular forces can effectively limit chain segment movement caused by thermal motion, thereby reducing thermal expansion. CTE value decreased the most at PI−1% film (CED 1% content). Although 1% CED content seemed small, it promoted chemical cross-linking between PI polymer chains. The increased crosslinking density enhanced the structural rigidity of the PI material, making it more stable upon thermal expansion. With the increase in CED content, PI−3% formed an interlocking stable structure with DPT molecules when small amounts of CE were added [52,53]. Then, it co-polymerized with diamine to form a network structure. With the increase in CE addition, a large number of free CED molecules existed in the solution, resulting in an increase in CTE. The CTEs of PI−5% and PI−7% are both 3 × 10^−6^ K^−1^, which is similar to inorganic materials such as low-temperature polycrystalline silicon (LTPS, 2.6 × 10^−6^ K^−1^) in flexible OLEDs.

### 3.4. Mechanical Properties

**Figure 5:**
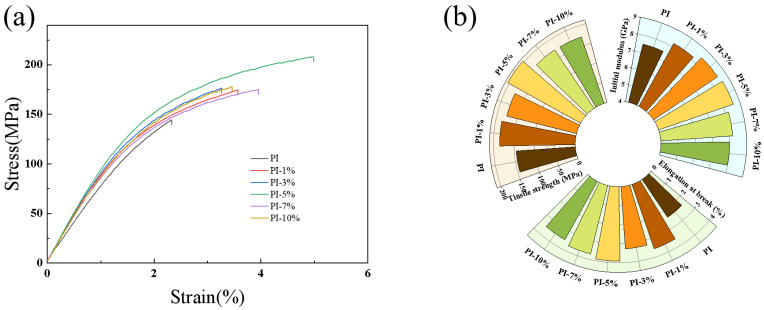
(**a**) Stress–strain curves of polyimide films, PI, PI−1%//PI−10%. (**b**) Mechanical data of polyimide films, PI, PI−1%//PI−10%.

Figure 5a shows typical stress–strain curves of the DPT/CED-BPDA PI films, and the corresponding data are listed in Figure 5b. The films exhibited excellent mechanical properties due to the presence of a biphenyl structure. The tensile modulus is 7.4 GPa, and the elongation at break can reach 2.4%. The elastic modulus of MPI films were improved after co-polymerization with CED, with a maximum tensile modulus of 8.6 GPa. The elongation at break and tensile strength first increased and then decreased. When a small amount of CED molecules was introduced into the PI molecular chain, the small amount of crosslinking increased the molecular chain spacing, resulting in a decrease in its mechanical properties [54]. With the increase in CED addition, more cross-linking structures formed between the PI molecular chains, which hindered the relative movement of PI molecular chains and made them more stable [55].

### 3.5. Dielectric Properties

**Figure 6:**
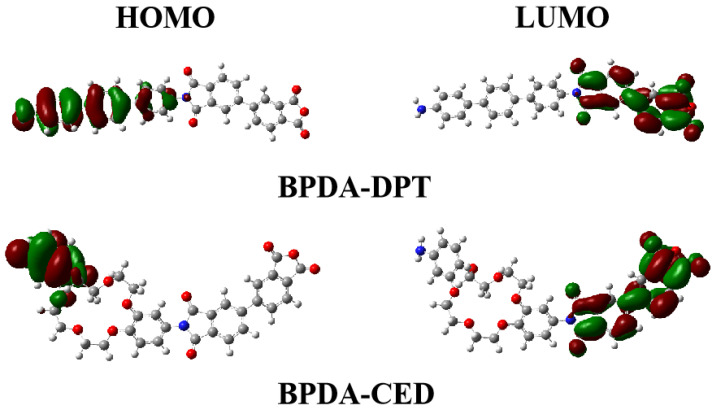
Distribution of electron clouds in HOMO and LUMO states of the molecular models of BPDA-DPT and BPDA-CED.

The energy levels of the PI/DPT and PI/CED PI films were calculated by the ground-state optimization structure in the highest electron-occupied orbital (HOMO) and lowest electron-occupied orbital (LUMO) states. The difference between HOMO and LUMO is called the energy level difference (ΔEg = LUMO − HOMO). As shown in Figure 6, the LUMO and HOMO of BPDA-DPT and BPDA-CED PI are −5.55 eV, −3.34 eV, and −5.47 eV and −3.34 eV, respectively, with ΔEg 2.21 eV and 2.13 eV. PI molecular chains can cross the CE rings and form a nested structure with CE molecules. The ΔEg value can be inferred to PI (BPDA-DPT) > PI (BPDA-CED/1%) > PI (BPDA-CED/3%) > PI (BPDA-CED/5%) > PI (BPDA-CED/7%) > PI (BPDA-CED/10%) > PI (BPDA-CED). The larger the ΔEg, the greater the energy barrier of the macromolecular conformation; additionally, it is more difficult to flip, which resulted greater rigidity in the PI molecular chains [56,57]. Therefore, the molecular chain rigidity of DPT/CED-BPDA ternary copolymerized PI lies between the two structural (BPDA-DPT and BPDA-CED) PIs.

**Figure 7:**
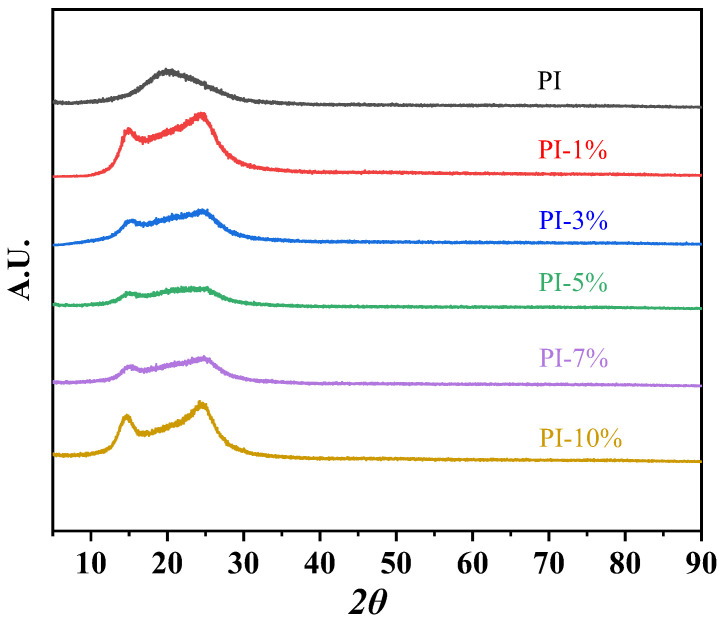
XRD curves of polyimide films, PI, PI−1%//PI−10%.

According to the XRD spectra in Figure 7, the amorphous diffraction peak of the prepared PI films can be observed to be at around 2θ = 20.0°. The molecular chain spacing was around 4.44 A, according to the Bragg’s formula (1) calculation. The peaks in the XRD map indicate the presence of some ordered structure in the sample. The peaks in the XRD map indicate the presence of ordered structures in the PI samples. PI materials were often considered to be amorphous or partially crystalline as high-performance polymers. The peaks at 15° and 25° indicate a local crystallization region and specific ordered structure due to the introduction of CEDs. CEDs promote certain arrangements of chain segments and form partially crystalline structures. The CEDs interact with PI polymer chains at the molecular level, guiding molecular chains to arrange in specific manner [48,49]. The common PI films are mainly amorphous, but the introduction of CED probably facilitates the formation of locally ordered structures, which appear as angle-specific diffraction peaks in the XRD map.

2dsinθ = nλ(1)

**Figure 8:**
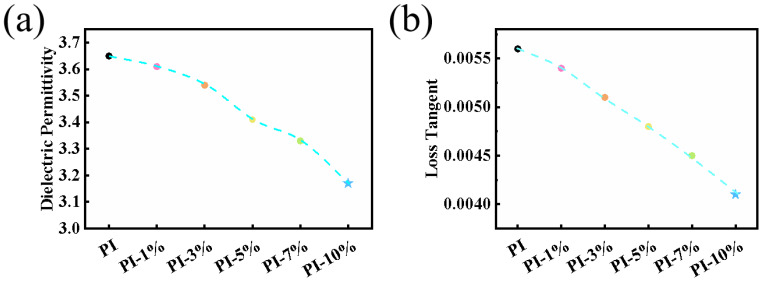
(**a**) Dielectric constant (D_k_) of polyimide films, PI, PI−1%//PI−10%. (**b**) Dielectric loss of polyimide film, PI, PI−1%//PI−10%.

The dielectric performances of PI/CED copolymerized films were tested at 10 GHz, and a volt–ampere diagram is shown in Figure 8a,b. It can be seen that the dielectric constants/losses of prepared PI films show the same change trend at 10 GHz. The dielectric constant decreased from 3.65 to 3.17, while the dielectric loss decreased from 5.6‰ to 4.1‰.

The introduction of crown ether leads to a reduction in all the PI samples’ overall polarity. The addition of crown ether also leads to a decrease in PI inter-molecular polar interactions and reduces the dielectric constant values. The large ring structure of the crown ether caused scattered distribution in the polar groups and reduced the overall polar effect of the PI material. The introduction of the crown ether may increase the motility of the PI molecular chains. The crown ether structure is more flexible compared to triphenyl structure, and this increased molecular mobility (flexibility) makes it difficult to achieve orientation polarization in an applied electric field. The increase in molecular motion makes it difficult for polar groups to effectively reorient during electric-field action, and it reduces the dielectric constant.

The dielectric constant, dielectric loss, and densities of different PI films were tested at 10 GHz, as shown in Table 3. The two positive correlation values of dielectric constant and density are 3.65 and 1.441 g/cm^3^, respectively. The dielectric constant and density of the PI/crown ether copolymerized films decreased after CED introduction. With the increase in CED, PI molecular chains added a crosslinking structure. Larger free volumes were given by CED cyclic macromolecules nested into the main PI molecular chains. The CED cross-linked molecules increased the PI molecular chain spacing due to their larger molecular size. The density and dielectric constant of PI−1%, PI−3%, PI−5%, PI−7% and PI−10% decreased to 1.406 g/cm^3^ and 3.61, 1.401 g/cm^3^ and 3.54, 1.395 g/cm^3^ and 3.41, 1.387 g/cm^3^ and 3.33, 1.382 g/cm^3^ and 3.17, respectively. Due to the lower inherent polarity of the benzene ring structure, the conjugation effects formation between benzene rings helps to uniformly distribute the PI electron density and reduce molecular polarization. The density and dielectric constant are positively correlated. The polarity is low due to the benzene ring structure in PIs. The formation of the conjugation effect between the benzene rings resulted in a uniform distribution of electron density, reducing the molecular polarization [8]. The density and dielectric constant decreased after the introduction of CED. The crosslinking inter-chain increased with the CED increase. The CE rings nested on the PI macromolecular chains, giving a larger free volume to the PI matrix [58,59,60]. When the amount of CED introduction increased, the number of PI molecular chains and chain crosslinking increased, while the nested PI−CED molecular structure further increased. Therefore, the density and dielectric constant of the PI−10% film are relatively low, at 1.382 g/cm^3^ and 3.17, respectively. The larger molecular spacing disrupted the dipoles rearranged in the electric field, reducing dielectric loss.

## 4. Conclusions

Diamine DPT and CED were synthesized and polymerized with BPDA to prepare PI films with low D_k_, high mechanical performances, and thermal stability. CED was introduced into the PI (BPDA-DPT) matrix to prepare corresponding ternary PI copolymerized films by in situ polymerization. The PI films exhibited good thermal performance, with Tg ranging from 371 °C to 391 °C, CTE -4 × 10^−6^ K^−1^ < CTE < 5 × 10^−6^ K^−1^, as the CED increased. The Young’s modulus could reach 8.6 GPa, and the tensile strength was above 200 MPa when the CED was introduced at 5% and 7%. The dielectric constant can be adjusted from 3.64 to 3.17, and the dielectric loss can be reduced from 5.6‰ to 4.1‰. Chuqi Shi et.al prepared PI composite membranes, ODA and PMDA, mixed with crown ether and sessiloxane (OAPrS) in their earlier research [61]. The dielectric constants were low, at 2.43–3.59, but their Young moduli were only about 5.89 GPa, the tensile strength values were about 146.8 MPa, and the Tg values were about 360.1 °C. The properties outside of dielectric properties are lower than the results of our work. This study reports a simple method of reducing PI films’ Dk and stabilizing their thermal and mechanical properties by introducing CED.

## Figures and Tables

**Table 1:** Experimental formulation of DPT/CED-BPDA polyimide.

System Name	n_(DPT)_^a^ (mmol)	n_(CED)_^a^ (mmol)	n_(BPDA)_^a^ (mmol)	Mass Fraction of _CED_(%) ^b^	Remark
PI (No CED)	15	/	15	/	film
PI−1% (1% CED)	14.85	0.15	15	1	film
PI−3% (3% CED)	14.55	0.45	15	3	film
PI−5% (5% CED)	14.25	0.75	15	5	film
PI−7% (7% CED)	13.95	1.05	15	7	film
PI−10% (10% CED)	13.50	1.50	15	10	film

^a^ Molar amount of DPT, CED, BPDA in PI film; ^b^ mass percentage of CED added in PI film.

**Table 2:** Thermal properties of the polyimides.

Sample	T_g_ (°C)	T_d5%_ (°C)	T_d10%_ (°C)	R_w750_	CTE (10^−6^ K^−1^)
PI	409	596	622	75.12	4
PI−1%	391	582	622	67.87	−4
PI−3%	407	575	618	67.94	−2
PI−5%	383	547	613	66.00	3
PI−7%	382	483	605	70.07	3
PI−10%	380	483	596	65.66	5

**Table 3:** Dielectric constant/loss and density of polyimide.

Sample	Dielectric Permittivity	Loss Tangent	Density (ρg/cm^3^)
PI	3.65	0.0056	1.441
PI−1%	3.61	0.0054	1.406
PI−3%	3.54	0.0051	1.401
PI−5%	3.41	0.0048	1.395
PI−7%	3.33	0.0045	1.387
PI−10%	3.17	0.0041	1.382

## Data Availability

Data are contained within the article.

## Supplementary Materials

The following supporting information can be downloaded at: https://www.mdpi.com/article/10.3390/polym16091188/s1, Figure S1: NMR spectra of DPT and CED.
