# Supplementary material for: Crown Ether Copolymerized Polyimide Film: Enhanced Mechanical, Thermal Properties and Low Dielectric Constant under High Frequency
Source: Polymers (Basel). 2024 Apr 24;16(9):1188. doi: 10.3390/polym16091188 (PMC11085621; doi:10.3390/polym16091188)
Supplement: Supplementary file 1 [file polymers-16-01188-s001.zip › polymers-2918201-supplementary.pdf]

1H NMR (600 MHz, DMSO-d6) δ 7.54 (s, 4H), 7.39 (d, J = 8.5 Hz, 4H), 6.66 (d, J = 8.5 Hz, 4H), 5.22 (s, 4H).

DPT

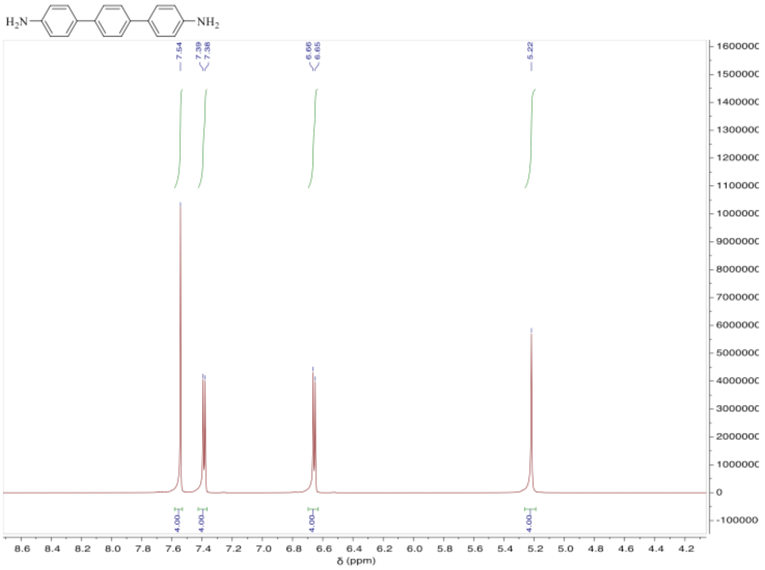

1H NMR (400 MHz, DMSO-d6) δ 6.63 (d, J = 8.4 Hz, 2H), 6.25 (s, 2H), 6.06 (d, J = 8.3 Hz, 2H), 4.64 (s, 4H), 3.99 – 3.91 (m, 8H), 3.83 – 3.75 (m, 8H).

CED

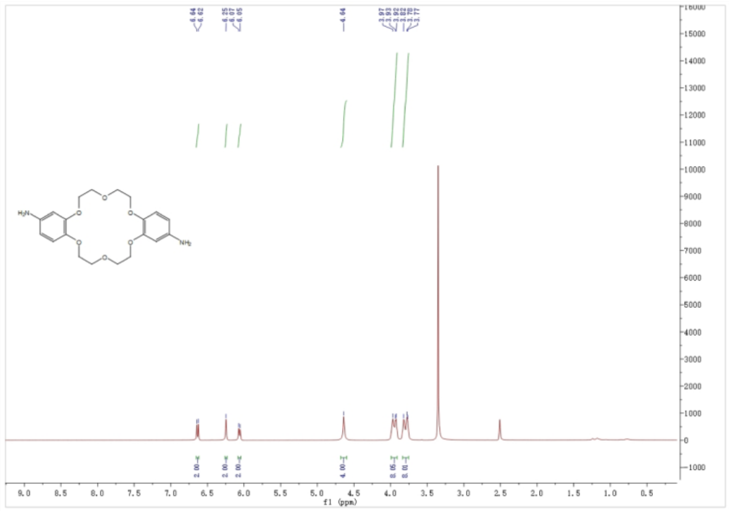

Figure S1. NMR spectra of DPT and CED.
